# Serum Biochemistry and Inflammatory Cytokines in Racing Endurance Sled Dogs With and Without Rhabdomyolysis

**DOI:** 10.3389/fvets.2018.00145

**Published:** 2018-07-19

**Authors:** Chris W. Frye, Sabine Mann, Jodie L. Joseph, Cristina Hansen, Brent Sass, Joseph J. Wakshlag

**Affiliations:** ^1^Department of Clinical Sciences, Cornell University College of Veterinary Medicine, Ithaca, NY, United States; ^2^Department of Population Medicine, Cornell University College of Veterinary Medicine, Ithaca, NY, United States; ^3^Veterinary Medicine, University of Alaska, Fairbanks, AK, United States; ^4^Wild and Free Kennels, Manley Hot Springs, Ithaca, AK, United States

**Keywords:** sled dog, rhabdomyolysis, potassium, phosphorus, creatine kinase

## Abstract

Serum muscle enzymes in endurance sled dogs peak within 2–4 days of racing. The object of this study was to compare mid-race serum chemistry profiles, select hormones, markers of inflammation, and the acute phase response in dogs that successfully completed half of the 2015 Yukon Quest sled dog race to their pre-racing samples (*n* = 14), as well as mid-race samples of successful dogs to those who developed clinical exertional rhabdomyolysis (**ER**) (*n* = 5). Concentrations of serum phosphorus in ER dogs were significantly elevated compared to healthy dogs (median 5.5 vs. 4.25 mg/dL, *P* < 0.01) at mid race. ALT, AST, and CK show a significant increase from pre-race baseline to mid-race chemistries (*P* < 0.01), with more pronounced increases in dogs with ER compared to healthy racing dogs (CK- median 46,125 vs. 1,743 U/L; *P* < 0.01). Potassium concentrations were significantly decreased from pre-race baselines in all dogs (median 5.1 vs. 4.5 mEq/L; *P* < 0.01), and even lower in dogs with ER (median 3.5 mEq/L; *P* < 0.01) mid-race. No changes in serum pro-inflammatory cytokine concentrations were noted in any groups of dogs. C-reactive protein was elevated in both groups of dogs, but significantly higher in those with ER compared with healthy dogs mid-race (median 308 vs. 164 ug/mL; *P* < 0.01). Healthy dogs may have CK elevations over 10,000 U/L, and dogs with ER were over 30,000 U/L. Although potassium decreases in healthy endurance sled dogs during racing, it remains in the normal laboratory reference range; however ER dog potassium levels drop further to the point of hypokalemia. Lastly increases in CRP may be reflective of a physiological response to exercise over the course of a race; however high CRP in ER dogs may be capturing an early acute phase response.

## Introduction

Ultramarathon sled dog racing induces physiological changes that are unique when compared to other canine sports. Such changes may be reflected on biochemistry profiles and include the presence of serum hyponatremia, hypoproteinemia as well as elevations in urea, alanine animotransferase (ALT), aspartate aminotransferase (AST), and creatine kinase (CK) (1–6). The enzyme CK plays an important role in creating energy substrate in various tissues. CK is abundant in muscle cells and serum elevations may reflect skeletal muscle damage (7, 8). CK elevations have been associated with exertional rhabdomyolysis (ER) in human exercise; however blood sampling and analysis of dogs during a clinical bout of exertional rhabdomyolysis has proven difficult.

Clinical features of ER may include myalgia, muscle spasm and stiffness, pigmenturia, and exertional collapse. The signs of disease are often disproportionately high for the amount of exercise performed. The pathophysiology of the disease may inherently involve multiple organ systems, leading to complications such as cardiac arrest, compartment syndrome, and acute renal failure. Early recognition and medical intervention improve the prognosis for ER and most dogs, like people, recover well (9, 10).

Previous canine literature has suggested that an elevation of CK >10,000 IU is supportive of ER and has been documented between days 2 and 4 of ultramarathon style sled dog racing (11). None of the dogs from the previous study were reported to have clinical signs of ER; therefore such elevations may reflect a range of physiological response to exercise as seen in people. Additionally certain serum cytokine concentrations (myokines) as well as c-reactive protein (CRP) are positively correlated with physiological muscle inflammation and damage during prolonged exercise in people; however they are less consistent in sled dogs (12–14). Horses with a history of ER show elevations of certain pro-inflammatory cytokines compared to a control group; however this has yet to be explored in dogs (15). Evidence of inflammation in dogs experiencing ER greater than an expected physiological response may reflect an underlying inflammatory disease or the beginnings of an acute phase response due to myonecrosis.

Although exertional rhabdomyolysis presents relatively commonly during ultramarathon racing and is a leading cause of mortality and morbidity in sled dogs (16, 17), little is known about its etiology. Multiple factors, alone or combined, may play a role in the development of ER and several have been observed in human medicine including electrolyte imbalances, underlying myopathies or genetic disorders, inflammatory disease, physical trauma, hyperthermia, poor conditioning, intense eccentric exercise, prescription drug use, and endocrine disorders (18, 19). Interestingly, both hyponatremia and hypokalemia have been implicated in the pathophysiology of human ER (18, 19) and both have been described in healthy ultra-marathon sled dogs (1, 3, 4, 6, 19–21).

The objective of this study was to compare canine serum biochemistry profiles, serum insulin, serum cortisol, select myokines, and CRP, of successful healthy dogs before racing and mid-race,as well as differences between successful dogs mid-race to dogs diagnosed with acute clinical ER. In addition, further regression analysis of serum electrolytes to serum CK status were also assessed.

## Materials and methods

### Animals

Dogs from three teams participating in the 2015 Yukon Quest endurance sled dog race from Whitehorse, Yukon Territory to Fairbanks, Alaska were solicited for blood collection at two time points. Immediately before the race and at mid race (mile 460) in Dawson City, Yukon during a mandatory 36 h rest period or at the time of drop for dogs diagnosed with ER. All procedures were approved by the Cornell University Institutional Care and Animal Use Committee and approval was sought from the Yukon Quest Board of Directors prior to the beginning of the study with informed and written consent of the owners.

### Blood collection

All dogs enrolled at the beginning of the study (*n* = 14) had a pre-racing sample taken approximately 36 h before the start of the race. In addition, dogs that developed ER were enrolled in the study as cases developed, but these animals did not have a baseline sample taken. Dogs were assessed for possible rhabdomyolysis at 4 checkpoints Braeburn (mile 100), Carmacks (mile 177), Pelly Crossing (mile 250), and Dawson City (mile 460). Case definition of ER included: collapse on the trail, or muscle cramping with evidence of visually assessed urine pigmenturia/myoglobinuria. Venipuncture of the cephalic vein was performed using a 20-gauge needle on each dog before fluid therapy was initiated and 10 ml of blood was collected. Serum was separated within 1 h of collection by centrifugation at 4,000 g for 6 min, transferred to separate tubes, immediately frozen on dry ice, and transported on dry ice to the investigators' laboratory.

### Serum biochemistry

Serum biochemical analysis included sodium, potassium, chloride, urea nitrogen, creatinine, calcium, phosphate, magnesium, total protein, albumin, globulin, alanine aminotransferase (ALT), aspartate aminotransferase (AST), and creatine kinase (CK), and was analyzed using a Olympus AU5400 automated analyzer (Olympus America, Center Valley, PA, USA) at the Cornell University Clinical Pathology Service.

### Serum cytokine assays

All samples from an individual dog were run on the same plate to eliminate inter-assay variability. A canine C - reactive protein kit (Tridelta PLC, Maynooth, Ireland) that has been validated for use on canine serum was used (13). The kit was used according to the manufacturers suggestions with all samples from the same dog being performed on the same plate in duplicate. All post exercise examples were dilute 1:10 as suggested by the manufacturer to ensure that concentrations fell within the linear portion of the standard curve (5–100 ug/ml). The canine electrochemoluminescent multiplexed cytokine kit [Proinflammatory Panel 3 (4-Plex)b; Mesoscale Discovery, Rockville, Maryland, USA] consisted of antibodies against canine TNF-α (inter-assay CV = 23.5%; intra-assay CV = 6.9%; LLOD = 0.17 pg/mL), IL-2 (interassay CV = 12.2%; intra-assay CV = 9.8%; LLOD = 7. pg/mL), IL-6 (inter-assay CV = 10.6%; intra-assay CV = 10.2%; LLOD = 2.4 pg/mL), IL-8 (inter-assay CV = 18.6%; intra-assay CV = 5.5%; LLOD = 1.3 pg/mL). Each sample from each sled dog was run in duplicate on the same plate, and a mean value was calculated based on standardized canine controls. All data were examined to assess whether the LLOD was reached. In the case that a LLOD was not met, and in an effort to avoid statistical bias, a value was placed on that missing data point as one half of the lower limit of detection (13).

### Serum insulin and cortisol assays

Serum insulin concentrations were measured with a commercially available human insulin radioimmunoassay (RIA) (EMD Millipore Corp, Billerica, MA). Serum insulin concentrations were validated for use on canine serum samples. Serial dilutions of 4 canine samples with assay buffer were parallel to the standard curve, and samples that were spiked with four different quantities of porcine insulin (Sigma-Aldrich, St. Louis, MO) had observed recoveries that averaged 94% of expected. The manufacturer did not report the cross-reactivity of the RIA antibody for canine insulin. The sensitivity of the assay, as reported by the manufacturer, is 2.72 μIU/mL. The mean intra- and inter-assay coefficients of variation were 4.3% and 7.3%, respectively. Serum cortisol was measured using a chemiluminescent assay (Siemens Cort-a-count Cortisol Kit, Siemens Corp. New York City, NY) that was previously validated for canine serum (22).

### Statistical analysis

Data was visually examined for normality and Shapiro Wilk testing was performed with many parameters not being normally distributed. In addition, the rhabdomyolysis group only contained 5 dogs making non-parametric testing appropriate. To account for the paired nature of the data for the analysis of the healthy dogs (*n* = 14), comparisons between pre-race and day 4 in the winning team of dogs were evaluated using Wilcoxon signed rank statistical analysis for each parameter (Graphpad Prism 6.0, La Jolla CA). For comparisons between day 4 results of the winning team and dogs with rhabdomyolysis (*n* = 5), a Mann Whitney *U*-test was performed for each parameter (Graphpad Prism 6.0, La Jolla CA) with an alpha set at 0.05. All results were adjusted for multiple comparisons using the Bonferroni method; therefore significance was set at *p* < 0.025.

Linear correlations were performed between the continuous variables CK, AST, potassium, sodium, chloride, phosphate, and magnesium in all 19 dogs during the mid-race/drop from race time point (Proc CORR, SAS 9.3, Cary, NC). To satisfy the assumption of normality, data for CK and AST were log transformed. The resulting correlation coefficient *r* was interpreted as follows regarding the relationship between two variables: *r* between 0 and 0.29/−0.29 = no linear association, between 0.30/−0.30 and 0.49/−0.49 = weak linear association; *r* between 0.50/−0.50 and 0.69/−0.69 = moderate linear association; *r* between 0.70/−0.70 and 0.99/−0.99 strong linear association.

## Results

### Dogs

The leading dog team had all 14 dogs from the team complete run race from Whitehorse, YK to Dawson City and consisted of 7 males and 7 females. Five dogs with ER were included in the study with all samples being collected between h 26–32 of racing at the Carmacks checkpoint, while the mid-race samples were collected at approximately h 76 of racing. The average age of the 14 dogs on the leading team was 4.2 ± 2.1 years old while the average age of the rhabdomyolysis dogs was 3.8 ± 0.8 years consisting of 2 males and 3 females.

### Comparing pre- and mid-race chemistries from the winning team

When examining the differences of serum chemistry parameters of the leading team before racing to mid-race, there were changes typical of ultramarathon sled dog racing with statistically significant increases in phosphorus, magnesium, and urea nitrogen (*P* < 0.01) concentrations, yet all values were within the reference range (Table 1). Serum enzyme differences at baseline compared to mid-race were also significantly elevated, including glucose (*P* < 0.01), serum ALT (*P* < 0.01), AST (*P* < 0.01), and CK (*P* < 0.01) with all dogs displaying values outside of the reference range for serum CK and AST (Table 1). Serum calcium, potassium, total protein, albumin, and creatinine at mid-race were significantly decreased from their baseline pre-race values, but within the reference ranges (*P* < 0.01; Table 1).

**Table 1:** Medians and ranges for serum biochemistry in dogs before and after successfully completing day 4 of 2015 Yukon Quest and 5 dogs with ER.

**Serum parameter**	**Reference range#**	**Day 0 healthy (*n* = 14)**	**Day 4 healthy (*n* = 14)**	**ER (*n* = 5)**	***p* (Day 0 vs. Day 4)**	***p* (Day 4 vs. ER)**
Na (mEq/L)	139–154	148 (145–151)	147 (144–150)	144 (143–148)	0.068	0.038
K (mEq/L)	3.6–5.5	5.1 (4.7–5.3)	4.5 (4.1–5.1)	3.5 (3.2–3.8)	0.001	< 0.001
Cl (mEq/L)	102–120	110 (107–115)	109 (103–112)	111 (107–113)	0.060	0.179
Ca (mg/dL)	8.9–11.4	10 (9.4–10.3)	8.8 (8.1–9.2)	8.8 (8.6–9.3)	< 0.001	0.626
P04 (mg/dL)	2.3–6.5	3.75 (3.2–4.4)	4.25 (3.8–5.4)	5.5 (5–5.9)	0.005	< 0.001
Mg (mEq/L)	1.5–2.5	1.7 (1.6–1.9)	1.9 (1.7–2)	1.9 (1.5–2)	0.001	0.682
SUN (mg/dL)	6–25	24.5 (17–36)	33 (27–38)	28 (23–70)	0.004	0.001
Creatinine (mg/dL)	0.5–1.6	0.6 (0.6–0.8)	0.5 (0.4–0.6)	0.6 (0.4–1.3)	0.001	0.500
Total Protein (g/dL)	5.0–7.4	6.2 (5.6–6.4)	5.2 (4.7–5.8)	4.8 (3.9–5.7)	< 0.001	0.428
Albumin (g/dL)	2.7–4.4	3.9 (3.6–4.1)	3.3 (3–3.6)	3.4 (2.4–3.6)	< 0.001	0.817
Glucose (mg/dL)	70–138	95.5 (68–115)	106 (90–134)	120 (105–160)	0.001	0.067
ALT (U/L)	5–107	73 (48–126)	150 (100–565)	246 (119–801)	< 0.001	0.074
AST (U/L)	5–55	30.5 (19–47)	53.3 (75–950)	2,305 (1,279–6,238)	< 0.001	< 0.001
CK (U/L)	59–895	148 (84–843)	1743.5 (771–18,222)	46,121 (30,600–137,198)	< 0.001	< 0.001

*P-value for differences from day 0 to day 4 derived from Wilcoxon signed rank test, those for difference between dogs successfully completing the race and dogs with exertional rhabdomyolysis (ER) using Mann-Whitney U-test. #Reference ranges established by Cornell University Diagnostic Laboratory Clinical Pathology as 5/95% confidence intervals for over 400 normal healthy dog samples*.

### Comparing mid-race chemistries from winning team to rhabdomyolysis dogs

**Figure 1:**
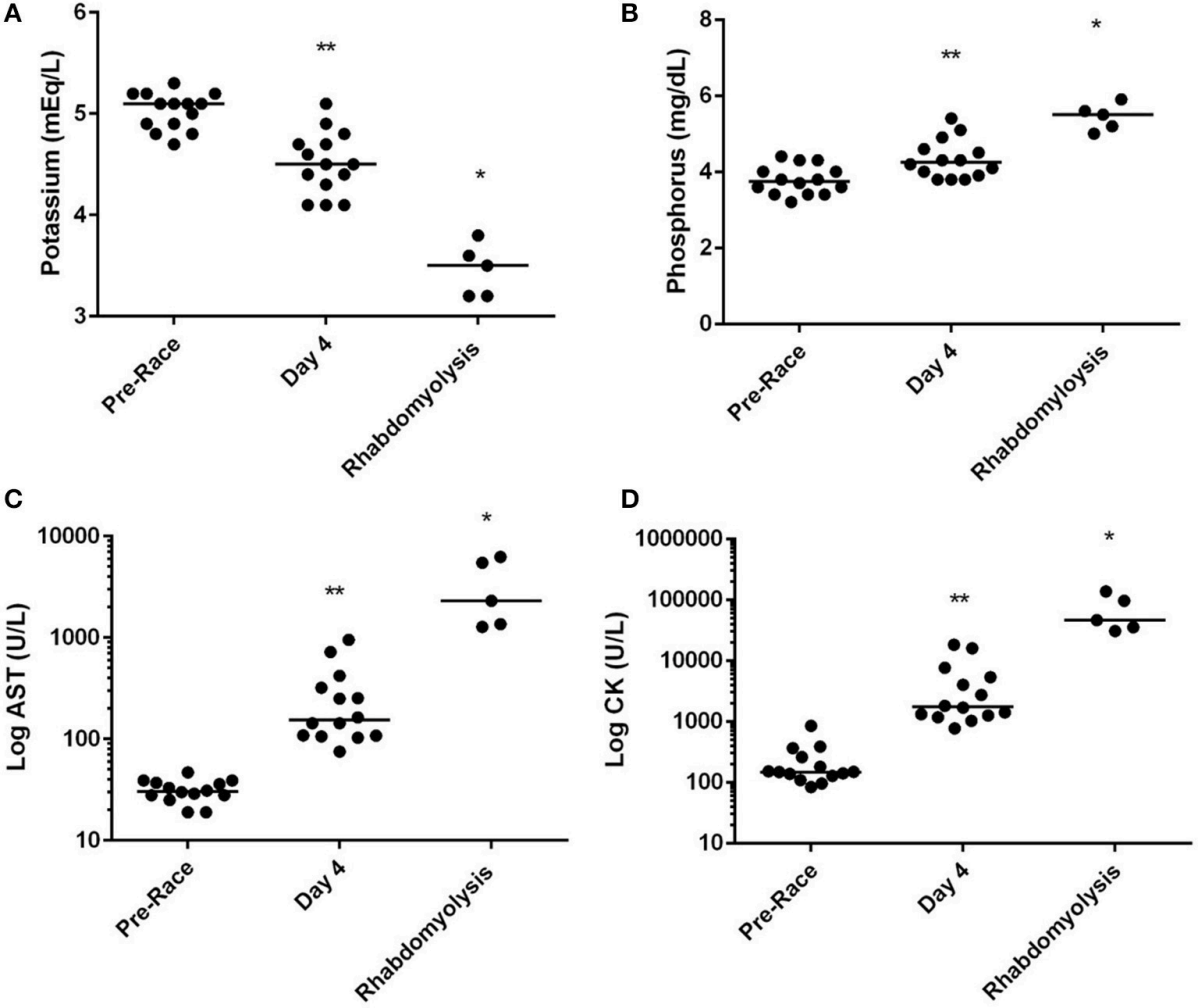
Median serum **(A)** potassium, **(B)** phosphorus, **(C)** log of aspartate amino transferase [AST] and log of creatine kinase [CK] concentration of successful racing dogs (*n* = 14) pre-racing and mid-race (Day 4), and dogs diagnosed with exertional rhabdomyolysis (*n* = 5) during the 2015 Yukon Quest. *Significant difference (*P* < 0.01) between day 4 and Rhabdomyolysis dogs. **Significant difference between pre-race and day 4 (*p* < 0.01).

Differences in electrolyte status were observed with concentrations of serum phosphorus in ER dogs being significantly elevated compared to dogs assessed at day 4 of racing (*P* < 0.01; Figure 1), while serum potassium (*P* < 0.01) was significantly decreased in ER dogs. CK and AST values were also significantly increased in ER dogs compared to the leading team dogs, while serum urea nitrogen was decreased (*P* < 0.01; Table 1; Figure 1).

### Correlations between CK, AST, and serum electrolytes

As the primary differences between successful dogs at mid-race and ER dogs were electrolyte and muscle enzyme changes; regression analyses were performed similar to Frank et al. (6) (Table 2). After log transformation for normality of serum CK and serum AST linear regression analysis showed that the rise in serum CK was negatively and moderately correlated with serum sodium (*r* = −0.50; *P* = 0.03) and showed a strong positive correlation with serum phosphorus concentrations (*r* = 0.80; *P* < 0.001). Serum potassium showed a strong negative correlation with serum CK (*r* = −0.74; *P* < 0.001). Both serum chloride and serum magnesium were not or only moderately correlated with serum CK (*r* < 0.34). Serum AST as a secondary marker of muscle membrane permeability showed very similar results as CK with moderate negative correlation to serum sodium (*r* = −0.55; *P* = 0.01) and strong correlations with serum phosphorus concentrations (*r* = 0.77; *P* < 0.001). Serum potassium showed a strong negative correlation with serum AST (*r* = −0.75; *P* < 0.001), and no or only moderate correlation with serum chloride or magnesium concentrations (*r* < 0.36).

**Table 2:** Pearson correlates between serum parameters of muscle cell permeability (Log CK, Log AST) and serum electrolytes (potassium, sodium, phosphorus, chloride, and magnesium) at mid race or at time of dropping from race for all dogs (*n* = 19).

**Variable**	**Log CK**	**Log AST**	**K**	**Na**	**Cl**	**PO4**	**Mg**
**Log CK**							
*r*	1.0	0.99	−0.74	−0.50	0.19	0.80	0.34
*P*		< 0.01	< 0.01	0.03	0.44	< 0.01	0.17
**Log AST**							
r		1.0	−0.75	−0.55	0.18	0.77	0.36
*P*			< 0.01	0.01	0.46	< 0.01	0.20
**Potassium**							
*r*			1.0	0.31	−0.47	−0.60	−0.47
*P*				0.20	0.06	0.01	0.07
**Sodium**							
*r*				1.0	0.20	−0.45	0.16
*P*					0.41	0.05	0.52
**Chloride**							
*r*					1.0	−0.12	0.12
*P*						0.63	0.64
**Phosphorus**							
*r*						1.0	0.34
*P*							0.17
**Magnesium**							
*r*							1.0

### Select myokines and C-reactive protein

None of the selected myokines (IL-2, IL-6, and IL-8, TNF-α) changed between the beginning and mid-race collections in the leading team, nor were there any differences between the values of the leading team and those with ER (Table 3). CRP (ug/mL) did significantly rise from a pre-race value of 21.9 (3.6–40.1) to a mid-race value of 164.2 (107.9–269.6) in the leading team (*p* < 0.01; Table 3). CRP was also significantly greater in the rhabdomyolysis dogs at 307.8 (217.5–486.4) when compared to the leading team at mid-race (*P* < 0.01; Table 3).

**Table 3:** Medians and ranges for selected cytokines, CRP, insulin and cortisol before (day 0) and after successfully completing day 4 of the 2015 Yukon Quest and 5 dogs with exertional rhabdomyolysis (ER).

	**Day 0 healthy (*n* = 14)**	**Day 4 healthy (*n* = 14)**	**ER (*n* = 5)**	***P* (day 0 vs. day 4)**	***P* (day 4 vs. ER)**
IL-2 (pg/mL)	837 (32–10,177)	413 (22–9,691)	570 (36–750)	0.26	0.57
IL-6 (pg/mL)	106 (14–891)	42 (13–1,810)	99(46–246)	0.67	0.50
IL-8 (pg/mL)	8,744 (1,656–28,078)	4,485 (549–24,031)	2,619 (1,251–1,634)	0.09	0.21
TNF-α (pg/mL)	2.5 (0.6–1432.1)	1.7 (0.6–1368.2)	31.3 (0.6–44.0)	0.65	0.73
CRP (μg/mL)	21.9 (3.6–40.1)	164.2 (107.9–269.6)	307.8 (217.5–486.4)	< 0.01	< 0.01
Insulin (uIU/mL)	28.3 (13.2–48.5)	12.8 (4.5–28.0)	11.3 (7.8–14.2)	0.02	0.47
Cortisol (ug/dL)	1.5 (1.0–2.5)	2.1 (1.0–5.8)	5.7 (2.8–14.4)	0.43	< 0.01

### Insulin and cortisol

Insulin decreased in the leading team from baseline to mid-race (28.3 vs. 12.8 IU/mL; *P* < 0.05); however there were no significant differences between the leading team at mid-race and the dogs with ER (12.8 vs. 11.3 IU/mL; *P* = 0.47; Table 3). Cortisol showed no changes over time between the pre-race values and mid-race values in the leading team; however the ER dogs had a significantly elevated cortisol compared to the leading team at the mid-race point (5.68 vs. 2.09 ug/dL; *P* < 0.01; Table 3).

## Discussion

Our study examined serum biochemistry, select hormones, and select myokines as well as measurements of acute phase response in order to compare dogs with clinical exertional rhabdomyolysis to those of the leading sled dog team in the 2015 1,000 mile Yukon Quest. The most notable differences to highlight between these groups are concentrations of serum creatine kinase, potassium, and phosphorous. Not only do these differences reflect the pathophysiology of ER, they may shed light onto the etiology of the disease, refine its laboratory definition, and help guide preventative measures. Most importantly, this defined electrolyte abnormality observed provides the nidus to examine potassium homeostasis in a more defined time frame within the first 2 days of racing which may include defined potassium intake, urinary losses as well as the hormonal milieu involved in sodium and potassium homeostasis.

Serum biochemistry changes have been previously examined during ultramarathon racing in a number of publications to date (1–4). Our findings are similar including a mild decrease in serum sodium and serum potassium (Table 1). Drops in serum calcium observed are expected, as total protein and albumin concentrations also decrease in dogs after running for 4 days (Table 1). The noted rises in serum urea nitrogen are attributed to being by-products of protein metabolism during high energy consumption and expenditure; however subclinical gastrointestinal ulceration cannot be ruled out (23, 24). In line with these demands, extreme stress on skeletal muscle occurs, which is reflected by abnormally high elevations in CK and AST values after racing for 4 days (Table 1). This alteration coincides with a similar peak observed by McKenzie and colleagues after 5 days of simulated racing; however, the slightly higher concentrations found in our dogs may be attributed to their level of competitiveness as they went on to win the 2015 Yukon Quest.

A CK value >10,000 U/L had been previously proposed as the laboratory cut off for consideration of rhabdomyolysis in ultramarathon sled dog racing (11). The degree of CK elevation during intense exercise in people does not correlate well to clinical exertional rhabdomyolysis and dramatic rises in CK can simply be part of the physiological response (18). Along these lines, 2 of the 14 healthy dogs examined 4 days in the race had CK concentrations in excess of 10,000 U/L, potentially reflecting their degree of work, genetics, and physiological adaptation rather than a pathological process.

Rhabdomyolysis was clinically confirmed in 5 dogs approximately 26–32 h into the race, and their serum CK values were all above 30,000 U/L with the highest value being over 125,000 U/L. These 5 dogs exhibited traditional signs of rhabdomyolysis including muscle cramping, short-strided gaits due to myalgia, and visually red/brown urine (myoglobin pigmenturia). Urine collected on these dogs showed no other abnormality other than positive hemoglobin when using an indicator strip. These data suggest that CK values likely need to be higher than 10,000 U/L to help confirm clinical suspicions of rhabdomyolysis within a population of canine athletes. Based on collective data including this study, a 50-fold rise above the upper limit of normal would be consistent with canine exertional rhabdomyolysis and interestingly has been proposed as a more specific laboratory cutoff for human diagnosis when compared to the traditionally more conservative cutoff of a 5-fold increase in CK from the upper limit of normal (18, 25).

The assessments of CK and timing of sampling are not without controversy as the half-life in dogs is approximately 2 h (7, 8). Our blood sampling occurred within an hour of clinical presentation at the checkpoints. In other words, all dogs ran into the checkpoint on their own volition with reports of poor performance, and no dog was discontinued from running prior to arrival at the checkpoint. This timing suggests little opportunity for CK values to diminish in the blood stream due to rest. Comparing the CK values from dogs successfully completing day 4 to those with confirmed exertional rhabdomyolysis is somewhat tenuous due to timing of sample collection; however the lack of differences except in muscle related enzymes, potassium, and phosphorus suggests an association between these electrolyte changes during rhabdomyolytic crisis. We may have missed the day 3 CK peak previously described by McKenzie and colleagues by taking our samples on the healthy dogs at day 4 (5). McKenzie and colleagues showed a 20% reduction in CK 1 day after peak levels; therefore, it is possible that CK values may have peaked at around 20,000 U/L in some of the 14 dogs successfully completing day 4 of racing. Piercy and colleagues have described a similar CK peak at day 3 occurring in a large population of Iditarod dogs that had been discontinued from racing due to various conditions (11). Our values coincide with these prior studies and demonstrate relative predictability of CK over time in the healthy endurance sled dog athlete.

Phosphorous, like CK, is released during muscle cell damage and is expected to result in serum concentration increases (26, 27). When examining serum phosphorous, ER dogs exhibited an elevation when compared to the leading team at day 4; however due to the nature of the study, no baseline values could be collected on ER dogs. No other changes on bloodwork or clinical exam could explain the increase in phosphorous within the ER group. The median phosphorous concentration of the leading team at Dawson City (4.2 meq/L) were within reference range and also similar to previous reports of endurance racing dogs in the similar racing conditions (2, 19). The increase in phosphorous in the ER dogs is unlikely attributable to any known physiological mechanism associated with exercise, particularly considering the strong correlation between serum CK and serum phosphorus observed. Normally phosphorous concentrations have been shown to mildly drop in similar racing conditions, as well as in shorter-duration moderate intensity exercise such as simulated search and rescue field work, hunting dogs and foxhounds which is attributed to increased glucose metabolism and metabolic demand of skeletal muscle during work (28–30). Mckenzie et al. (3) did not show any significant changes in phosphorous over time in a simulated 500 mile ultramarathon while taking blood samples every 100 miles. Based on these previous studies, it is reasonable to expect no change or a physiological decrease in phosphorous in exercising sled dogs running. Therefore the rises noted in the ER dogs are most likely attributed to pathophysiology of muscle breakdown.

When comparing the ER dogs to the leading team at mid-race, another dramatic and unexpected finding was noted in potassium concentrations which were strongly negatively correlated to the rise in serum CK. The ER dogs exhibited hypokalemia with a mean of 3.5 meq/L which is both counter intuitive (as potassium is released from the intracellular space into the serum when the sarcolemma is compromised) and contrary to consistently published results of hyperkalemia in people presenting with ER (18). Both insulin and cortisol were examined as influential hormones on serum potassium homeostasis with insulin driving potassium intracellularly and cortisol acting as a counter-regulatory hormone (31). Insulin was significantly lower in the mid-race samples as compared to the pre-race samples from the same dogs, but no difference was noted between the mid-race dogs and those with ER. Cortisol was elevated in the rhabdomyolysis dogs compared to the healthy mid-race dogs, but did not change in the healthy dogs from the beginning of the race to the midpoint. Previous data on ultramarathon dog racing is consistent with unchanged cortisol levels (2). Based on this data, hormone influence over potassium could not account for lower serum concentrations. The lower insulin levels and higher cortisol levels are expected during intense exercise to maintain serum glucose and have been previously described (2, 31). In fact, a significant mild rise in glucose was noted over time in the healthy dogs, and was higher in the ER dogs compared to the healthy dogs at day four. Glucose elevations have been previously reported as a parameter that is altered in marathon or ultramarathon racing sled dogs (1–4). The modest increase by day 4 of racing may be related to timing or sampling as it relates to feeding as this was not controlled for. The modest, nearly significant increase in dogs with rhabdomyolysis is likely related to the stress of the disease process and may be subsequent to increased cortisol concentrations (32).

It stands to reason that prior to muscle cell compromise in our ER dogs, the serum potassium may have been even lower than 3.5 meq/L, however we cannot rule out the ER may somehow result in increased potassium clearance. If intracellular potassium release during ER failed to elevate serum potassium into or above the normal range, it is reasonably to speculate that a whole-body potassium depletion was present in dogs prior to presenting with acute ER. Yet the question remains: what are the mechanisms for potassium depletion in ER dogs?

Electrolyte status in endurance sled dogs has been studied extensively to try to understand the mild decrease in serum sodium and potassium that is often observed during racing (1, 2, 4); however, not all studies have shown decreased serum sodium (3). Seminal studies by Hinchcliff and colleagues suggest that the increased water turnover in these dogs leads to mild urinary sodium losses and upregulation of the renin-angiotensin aldosterone system for sodium conservation (19, 20). This response may lead to a modest hypokalemia as a physiological tradeoff in the face of sodium retention. Further supporting this proposed mechanism, providing approximately 1000 mg/1000 kcals of sodium to a team of racing dogs prevented the typical decrease in both serum sodium and potassium previously described compared to two unsupplemented teams (4). As of now, electrolyte supplementation in dogs has not been thoroughly investigated since oral supplements have been associated with gastroenteritis (33).

Electrolyte disturbances including hypokalemia due to global potassium depletion have been proposed as an underlying mechanism for ER in people using dog models (34). Whole-body potassium depletion was achieved through dietary restriction of the electrolyte in an experimental group of dogs compared to a control group. Potassium depletion reflected in part by a serum concentration range of (1.8–3.3 mEq/L) predisposed dogs to local ischemia during repetitive muscle contraction mimicking intense exercise resulting in myonecrosis and ER. The potassium serum range of the ER dogs was 3.2–3.8 mEq/L and may have been lower prior to sarcolemma disruption during rhabdomyolysis. It is possible that a low local potassium concentration could predispose these dogs to ER within certain muscle groups depending on the intensity they were exercised relative to other muscles.

Lastly, select pro-inflammatory cytokines and the acute phase response as represented by CRP were compared between ER dogs and healthy mid-race athletes. There were no changes in the select cytokine group (IL-2, IL-6, IL-8, TNF-α) in the healthy dogs and no differences between them and those affected by ER. Such findings are consistent with previous sled dog studies and are contrary to elevations of such signaling molecules in human ultramarathon racing (12, 13). The CRP was elevated in both groups of dogs, but higher amongst those with ER at a mean of 307 ug/mL which is on par with prior measurements in endurance sled dogs for a 346-mile race (35). The difference between groups could simply be attributed to timing of the sample acquisition or an early acute phase inflammatory response to myonecrosis on top of a normal physiological response to exercise. The ER dogs were sampled within 32 h of racing which is much earlier than other endurance racing studies suggesting that the acute phase increase in sled dogs is relatively early (13, 35). In these other studies where dogs were assessed well into ultra-marathon racing it is noted that concentrations tend to slowly diminish over time. Whether the increases we have observed in ER dogs are just part of the normal physiological response early in marathon racing or a possible heightened inflammatory response in these dogs cannot be determined due to the differences in timing of sampling between the ER dogs and the successful dogs at mid-race. The timing of the inflammatory response to exercise is still poorly defined in sled dogs and there is a need for sequential daily CRP assessment in racing situations to fully understand the dynamics of CRP, yet we speculate that there is an early elevation that diminishes over time during racing suggesting no egregious inflammatory response as part of the ER crisis, but further controlled field studies are needed.

Overall our study has limitations including lack of a true control group of dogs, small sample size, and an inability to examine dietary patterns of the dogs in question. Given the nature of competitive endurance sled dog racing and the unpredictability of dogs developing clinical rhabdomyolysis, a true control group would be difficult to assemble. Therefore it becomes prudent to partially rely on the consistency of previous literature in helping to explain this phenomena or qualify differences noted amongst the comparable variables. Additionally, using the winning team for comparison seems logical as they should have the most dramatic physiological responses and demands in which to compare an exertional disease process and share similarities in race conditions. Until the ideal study can be done following large numbers of dogs collecting daily blood and urine samples with appropriate dietary intake assessment we must rely on small numbers in field studies to better understand the physiological responses related to ER in sled dogs.

## Conclusions

Despite some limitations, the data collected during this study comparing healthy sled dogs that won the race and those that developed clinical rhabdomyolysis is unique. Such findings may help establish more appropriate guidelines for serum CK values in confirming a diagnosis of the disease. In addition, the electrolyte changes found, particularly lower serum potassium, may be related to a pathophysiological mechanism of ER. Questions regarding supplementing potassium in the diet or, possibly, sodium (to diminish the aldosterone response for sodium conservation) for prevention of rhabdomyolysis remains unanswered, warranting further investigation with a more targeted approach with appropriate timing of sampling. Underlying inflammatory disease seems to be an unlikely contributor to developing ER and differences in C-reactive protein between ER dogs and healthy dogs more likely reflect an early acute phase response to muscle damage that may be related to timing of sample collection.

## Author contributions

CF, JW, CH, and BS postulated the experimental design. JW, CF, and BS performed work associated with this study. JW, CF, CH, and SM performed statistical analysis and prepared manuscript. All authors reviewed manuscript upon submission.

### Conflict of interest statement

The authors declare that the research was conducted in the absence of any commercial or financial relationships that could be construed as a potential conflict of interest.

## References

[1] Burr JR Reinhart GA Swenson RS Swaim SF. Serum biochemical values in sled dogs before and after competing in long-distance races. J Am Vet Med Assoc. (1997) 211:175–9. 9227746

[2] Hinchliff KW Olson J Crusberg C Kenyon J Long R Royle W Serum biochemical changes in dogs competing in a long-distance sled race. J Am Vet Med Assoc. (1993) 202:401–5. 8440630

[3] McKenzie EC Jose-Cunilleras E Hinchcliff KW Holbrook TC Royer C Payton ME Serum chemistry alterations in Alaskan sled dogs during five successive days of prolonged endurance exercise. J Am Vet Med Assoc. (2007) 23:1486–92. 10.2460/javma.230.10.1486 17504039

[4] Ermon V Yazwinski M Milizio JG Wakshlag JJ. Serum chemistry and electrolyte alterations in sled dogs before and after a 1600 km race: dietary sodium and hyponatraemia. J Nutr Sci. (2014) 3:1–5. 10.1017/jns.2014.39 26101595 PMC4473165

[5] Wakshlag JJ Snedden K Reynolds AJ. Biochemical and metabolic changes due to exercise in sprint-racing sled dogs: implications for postexercise carbohydrate supplements and hydration management. Vet Ther. (2004) 5:52–9. 15150730

[6] Frank L Mann S Johnson J Downey RL Wakshlag JJ Plasma chemistry before and after two consecutive days of racing in sled dogs: associations between muscle damage and electrolyte status. Comp Ex Phys. (2015) 11:151–8. 10.3920/CEP150020

[7] Aktas M Lefebvre HP Toutain PL Braun JP Disposition of creatine kinase activity in dog plasma following intravenous and intramuscular injection of skeletal muscle homogenates. J Vet Pharm Ther. (1995) 8:1–6. 10.1111/j.1365-2885.1995.tb00542.x 7752299

[8] Aktas M Auguste D Lefebvre HP Toutain PL Braun JP. Creatine kinase in the dog: a review. Vet Res Comm. (1993) 17:353–69. 10.1007/BF01839386 8209415

[9] Cervellin G Comelli I Lippi G. Rhabdomyolysis: historical background, clinical, diagnostic and therapeutic features. Clin Chem Lab Med. (2010) 48:749–56. 10.1515/CCLM.2010.151 20298139

[10] Poels PJ Gabreëls FJ. Rhabdomyolysis: a review of the literature. Clin Neuro Neurosurg. (1993) 95:175–92. 10.1016/0303-8467(93)90122-W 8242960

[11] Piercy RJ Hinchcliff KW Morley PS DiSilvestro RA Reinhart GA Nelson SL Jr. Vitamin E and exertional rhabdomyolysis during endurance sled dog racing. Neuromuscular Disord. (2001) 11:278–86. 10.1016/S0960-8966(00)00199-1 11297943

[12] Kasapis C Thompson PD. The effects of physical activity on serum C-reactive protein and inflammatory markers: a systematic review. J Am Coll Cardiol. (2005) 45:1563–9. 10.1016/j.jacc.2004.12.077 15893167

[13] Yazwinski M Milizio JG Wakshlag JJ. Assessment of serum myokines and markers of inflammation associated with exercise in endurance racing sled dogs. J Vet Int Med. (2013) 27:3716. 10.1111/jvim.12046 23398265

[14] von Pfeil DJ Cummings BP Loftus JP Levine CB Mann S Downey RL Evaluation of plasma inflammatory cytokine concentrations in racing sled dogs. Can Vet J. (2015) 56:1252. PMC4668826 26663920

[15] El-Deeb WM El-Bahr SM. Investigation of selected biochemical indicators of Equine Rhabdomyolysis in Arabian horses: pro-inflammatory cytokines and oxidative stress markers. Vet Res Comm. (2010)34:677–89. 10.1007/s11259-010-9439-5 20830520

[16] Dennis MM Nelson SN Cantor GH Mosier DA Blake JE Basaraba RJ. Assessment of necropsy findings in sled dogs that died during Iditarod Trail sled dog races: 23 cases (1994-2006). J Am Vet Med Assoc. (2008) 232:564–73. 10.2460/javma.232.4.564 18279094

[17] Long RD Treatment of common injuries in endurance racing sled dogs. Compendium (1993) 15:434–7.

[18] Scalco RS Snoeck M Quinlivan R Treves S Laforét P Jungbluth H. Exertional rhabdomyolysis: physiological response or manifestation of an underlying myopathy? BMJ Open Sport Exer Med. (2016) 2:e000151. 10.1136/bmjsem-2016-000151 27900193 PMC5117086

[19] Szczepanik ME Heled Y Capacchione J Campbell W Deuster P O'Connor FG. Exertional rhabdomyolysis: identification and evaluation of the athlete at risk for recurrence. Curr Sports Med Rep. (2014) 13:113–9. 10.1249/JSR.0000000000000040 24614425

[20] Hinchcliff KW Reinhart GA Burr JR Schreier CJ Swenson RA. Effect of racing on serum sodium and potassium concentrations and acid-base status of Alaskan sled dogs. J Am Vet Med Assoc. (1997) 210:1615–8. 9170088

[21] Hinchcliff KW Reinhart GA Burr JA Swenson RA. Exercise-associated hyponatremia in Alaskan sled dogs: urinary and hormonal responses. J Appl Phys. (1997) 83:824–9. 10.1152/jappl.1997.83.3.824 9292469

[22] Singh AK Jiang Y White T Spassova D. Validation of a chemiluminescent immunoassay methods for the analysis of thyroxine and cortisol in blood samples obtained from dogs, cats and horses. J Vet Diag Invest. (1997) 9:261–8. 10.1177/104063879700900307 9249165

[23] Ritchey JW Davis MS Breshears MA Willard MD Williamson KK Royer CM. Gastritis in Alaskan racing sled dogs. J Comp Pathol. (2011) 145:68–76. 10.1016/j.jcpa.2010.11.008 21247587

[24] Davis M Willard M Williamson K Royer C Payton M Steiner JM. Temporal relationship between gastrointestinal protein loss, gastric ulceration or erosion, and strenuous exercise in racing Alaskan sled dogs. J Vet Intern Med. (2006) 20:835–9. 10.1111/j.1939-1676.2006.tb01794.x 16955805

[25] Kenney K Landau ME Gonzalez RS Hundertmark J O'Brien K Campbell WW. Serum creatine kinase after exercise: drawing the line between physiological response and exertional rhabdomyolysis. Muscle Nerve. (2012) 45:356–62. 10.1002/mus.22317 22334169

[26] Gabow PA Kaehny WD Kelleher SP. The spectrum of rhabdomyolysis. Medicine. (1982) 61:141–52. 10.1097/00005792-198205000-00002 7078398

[27] Chatzizisis YS Misirli G Hatzitolios AI Giannoglou GD. The syndrome of rhabdomyolysis: complications and treatment. Eur J Int Med. (2008)19:568–74. 10.1016/j.ejim.2007.06.037 19046720

[28] Davenport GM Kelley RL Altom EK Lepine AJ. Effect of diet on hunting performance of English Pointers. Vet Ther. (2001) 2:10–23. 19753695

[29] Spoo JW Zoran DL Downey RL Bischoff K Wakshlag JJ. Serum biochemical, blood gas and antioxidant status in search and rescue dogs before and after simulated fieldwork. Vet J. (2015)206:47–53. 10.1016/j.tvjl.2015.07.002 26228710

[30] Huntingford JL Kirn BN Cramer K Mann S Wakshlag JJ. Evaluation of a performance enhancing supplement in American Foxhounds during eventing. J Nutr Sci. (2014)3:e24. 10.1017/jns.2014.38 26101593 PMC4473135

[31] Wasserman DH Lickley HL Vranic M. Interactions between glucagon and other counterregulatory hormones during normoglycemic and hypoglycemic exercise in dogs. J Clin Invest. (1984) 74:1404–14. 10.1172/JCI111551 6148356 PMC425308

[32] Angle CT Wakshlag JJ Gillette RL Stokol T Geske S Adkins TO. Hematologic, serum biochemical, and cortisol changes associated with anticipation of exercise and short duration high-intensity exercise in sled dogs. Vet Clin Pathol. (2009) 38:370–4. 10.1111/j.1939-165X.2009.00122.x 19351341

[33] Young DR Iacovina A Erve P Mosher R Spector H. Effect of time after feeding and carbohydrate or water supplement on work in dogs. J Appl Phys. (1959) 14:13–7. 10.1152/jappl.1959.14.6.1013 13846615

[34] Knochel JP Schlein EM. On the mechanism of rhabdomyolysis in potassium depletion. J Clin Invest. (1972) 51:1750–8. 10.1172/JCI106976 5032523 PMC292322

[35] Wakshlag JJ Stokol T Geske SM Greger CE Angle CT Gillette RL. Evaluation of exercise-induced changes in concentrations of C-reactive protein and serum biochemical values in sled dogs completing a long-distance endurance race. Am J Vet Res. (2010) 71:1207–13. 10.2460/ajvr.71.10.1207 20919909

